# Microfluidic Device Directly Fabricated on Screen-Printed Electrodes for Ultrasensitive Electrochemical Sensing of PSA

**DOI:** 10.1186/s11671-019-2857-6

**Published:** 2019-02-28

**Authors:** Shouhui Chen, Zhihua Wang, Xinyuan Cui, Linlei Jiang, Yuee Zhi, Xianting Ding, Zhihong Nie, Pei Zhou, Daxiang Cui

**Affiliations:** 1 Center of Food Safety Engineering and Technology Research, Shanghai, Key Laboratory of Urban Agriculture, Ministry of Agriculture, School of Agriculture and Biology, Shanghai Jiao Tong University, 800 Dongchuan Road, Shanghai, 200240 China; 2 Institute of Nano Biomedicine and Engineering, Shanghai Engineering Research Center for Intelligent Instrument for Diagnosis and Therapy, Key Lab. for Thin Film and Microfabrication Technology of Ministry of Education, Department of Instrument Science and Engineering, School of Electronic Information and Electrical Engineering, Shanghai Jiao Tong University, 800 Dongchuan Road, Shanghai, 200240 China; 3 Department of Medical Imaging, Second Clinical College of Chongqing Medical University, No.74 Linjiang Road, Yuzhong District, Chongqing, 400016 China; 4 Institute for Personalized Medicine, School of Biomedical Engineering, Shanghai Jiao Tong University, Shanghai, 200030 China; 5 Department of Chemistry and Biochemistry, University of Maryland, College Park, MD 20742 USA; 6 National Center for Translational Medicine, Collaborative Innovational Center for System Biology, Shanghai Jiao Tong University, 800 Dongchuan Road, Shanghai, 200240 China

**Keywords:** Screen-printed electrode, Microfluidic devices, PSA, Electrochemical sensor, Detection

## Abstract

How to fabricate scale low-cost microfluidic device for detection of biomarkers owns a great requirement. Herein, it is for the first time reported that a new microfluidic device based on bonding polydimethylsiloxane microfluidic channels onto the substrate of a screen-printed electrode with coating glass solution was fabricated for electrochemical sensing of prostate-specific antigen (PSA). Compared to traditional microfabrication processes, this method is simple, fast, low cost, and also suitable for mass production. The prepared screen-printed electrode-based microfluidic device (CASPE-MFD) was used for the detection of the PSA in human serum. The prepared CASPE-MFD had a detection limit of 0.84 pg/mL (25.8 fM) and a good linearity with PSA concentration ranging from 0.001 to 10 ng/mL, which showed a great promise platform toward the development of miniaturized, low-cost electrochemical microfluidic device for use in human health, environmental monitoring, and other applications.

**Electronic supplementary material:**

The online version of this article (10.1186/s11671-019-2857-6) contains supplementary material, which is available to authorized users.

## Background

Microfluidic system is the process of manipulation of fluids of small volume (10^−9^ to 10^−18^ L) within channels with a dimension of tens to hundreds of micrometers [1]. This technology has shown great potential in biomedicine, environmental monitoring, and food safety analysis. In particular, microfluidic devices (MFDs) typically exhibit the following advantages, including small footprints, reduced consumption of reagents, multiple sample detection in parallel, increased reliability, sensitivity, and high and large-scale integration [2–4].

Electrochemical sensors have been widely integrated and hyphenated with sampling, fluidic handling, separation, and other engineering detection scenarios [5]. The application of electrochemical sensors for biomolecule detection is promising since electrochemical sensors exhibit numerous advantages such as high sensitivity and selectivity, reliable reproducibility, simple use for continuous on-site analysis, minimal sample preparation, relatively low cost, and short-time response. Electrochemical system can be easily integrated within a microfluidic system [6, 7], and this offers advantages over a conventional analytical platform [8–10], such as ease in sample preparation, excellent sensitivity and versatility, and the removal of bulky optical components [11, 12].

**Fig. 1:**
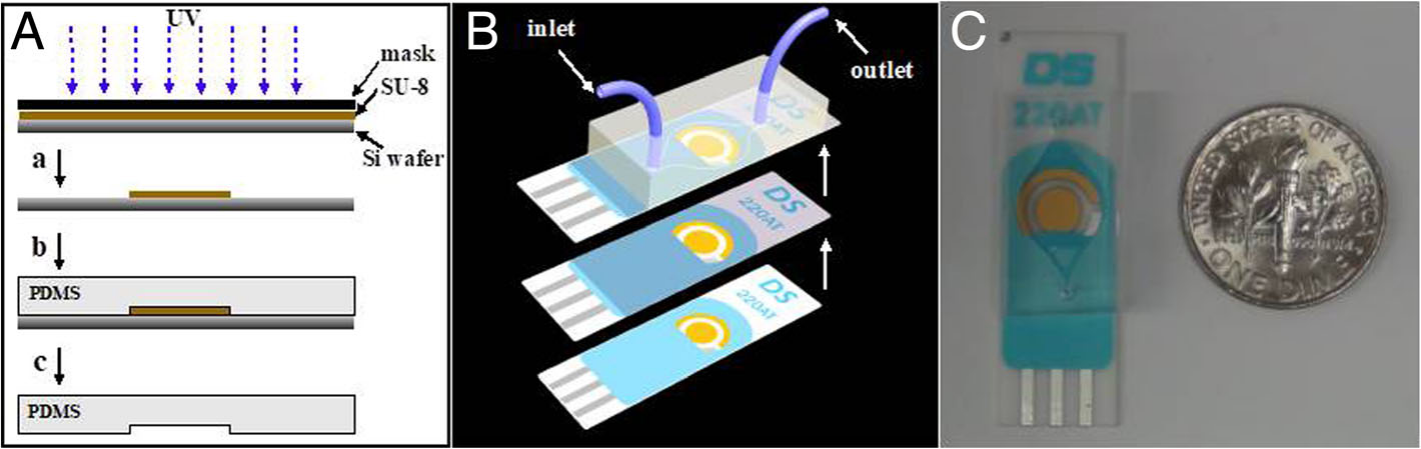
**a** Fabrication process for the PDMS microfluidic channels patterned by SU-8 photolithography. **b** Fabrication process for the commercially available screen-printed electrode-based microfluidic device. The CASPE-MFD comprises PDMS microfluidic channels, two printed gold electrodes as the working and counter electrodes, and a printed silver electrode as the pseudo-reference electrode. **c** A commercially available screen-printed electrode-based microfluidic device

In this study, a simple, inexpensive, and versatile strategy was used for the fabrication of electrochemical sensing MFDs using commercially available screen-printed electrodes for point-of-care diagnosis. The developed device was defined as CASPE-MFDs (commercially available screen-printed electrode-based microfluidic devices). The polydimethylsiloxane (PDMS) microfluidic channels were firstly patterned using standard photolithography, and the CASPE-MFDs were fabricated by directly bonding PDMS microfluidic channels on a commercially available screen-printed electrode (Fig. 1). The screen-printed electrode was directly used and coated by a thin layer of glass using sol-gel approach [13]. Subsequently, PDMS microfluidic channels were bonded onto the electrode after plasma treatment of their surfaces. The CASPE-MFDs are capable of quantifying the concentration of various analytes in biological fluids such as phosphate buffer solution (PBS) and serum samples. The CASPE-MFDs were used to demonstrate the detection and quantification of prostate-specific antigen (PSA) biomarker in PBS buffer solutions and human serum samples using chronoamperometry (CA) and square wave voltammetry (SWV). The detection of PSA in this device showed a high sensitivity, and the limit of detection (LOD) for PSA is 0.84 pg/mL (25.8 fM). The LOD is over 100 times more sensitive than the 0.1 ng/mL clinical limit of detection for commercial assays [14] and better than other devices [3, 15, 16]. The CASPE-MFD is portable, is simple to use, and has the potential to integrate other components such as sample preparation and separation systems.

## Materials and Methods

### Chemical Reagents and Materials

Prostate-specific antigen (PSA) and multiclonal anti-PSA antibody horseradish peroxidase (HRP) were purchased from Petsec Energy Ltd. Biotinylated anti-PSA antibody, streptavidin magnetic beads, bovine serum albumin, and hydroquinone were from Fisher Scientific. Tween-20, hydrogen peroxide (H_2_O_2_; 30%), and ferrocenecarboxylic acid were from Sigma-Aldrich. SU-8 2075 was from MicroChem Corp. The polydimethylsiloxane (PDMS) pre-polymer and curing agent were purchased from Dow Corning. All immunoreagents were dissolved in 1× pH 7.4 PBS buffer solutions from KD Medical Solutions. All chemical reagents were prepared with ultrapure water from a Millipore Milli-Q water purification system.

### Instrumentation

The fluorescence microscope was performed on an Olympus U-CMAD3 (Olympus, Japan). The μCSPE devices were fabricated by a Plasma cleaner PDC-32G (Harrick Plasma, USA). All electrochemical measurements were performed by CHI 760B (CHI, China) with a conventional three-electrode system, which consists of two printed gold electrodes as working and counter electrode, respectively, and a printed silver electrode as pseudo-reference electrode (Fig. 1).

### Microfluidic Chip Fabrication

The PDMS microfluidic channels were patterned using standard photolithography. Briefly, a silicon wafer, rinsed with a mixed solution (H_2_SO_4_/H_2_O_2_ = 7/3) followed by ultrapure water clean, was coated with SU-8 2075 photoresist. The wafer was then baked at 65 °C for 7 min followed by 95 °C for 40 min to remove solvents and photo-exposed to UV light for 15 s through a photomask. The whole system was baked at 65 °C for 5 min followed by 95 °C for 15 min to stabilize the polymerization. The unpolymerized photoresist was removed by soaking the silicon wafer in SU-8 developer and washing with isopropanol and deionized water. The mixtures of PDMS pre-polymer solution and curing agent (10,1) were casted over the pre-described silicon wafer, cured at 65 °C for 2 h, and peeled off [17].

The commercially available printed electrode was coated with a layer of glass using sol-gel approach. Briefly, tetra ethoxy silane (TEOS), MTES, ethanol, and water were fully mixed at a proportion of 1:1:1:1 and sonicated for 5 min. The mixtures were placed in an oven at 65 °C overnight. The electrode was placed on a hot plate for 5 min at 80 °C before glass coating and then smeared with the precursor mixtures using a brush to avoid the mixtures invading into the electrode surface. The electrode was dried at room temperature after the smearing. The PDMS chip and glass-covered electrode were then processed with O_2_ plasma for 30 s and adhered to each other.

### Chronoamperozmetric Experiments

Chronoamperometric experiments were carried out in 1× pH 7.4 PBS containing 4.5 mM hydroquinone and 0.1 mM hydrogen peroxide solutions at a − 2.0 mV step potential (vs. a silver pseudo-reference electrode) and generated the calibration curve for the concentration of PSA from 0 to 10 ng mL^−1^. Briefly, we injected 50 μL of 0.2 mg mL^−1^ magnetic bead-conjugated anti-PSA antibody to μCSPE devices at the rate of 50 μL min^−1^, and washed thoroughly using 100 μL pH 7.4 PBS at the rate of 50 μL min^−1^. Besides, 50 μL of a blocking buffer (0.05% (*v*/*v*) Tween-20 and 2% (*w*/*v*) bovine serum albumin (BSA) in PBS) was injected at the rate of 10 μL min^−1^ and incubated for 30 min under 37 °C condition, washed thoroughly using 100 μL pH 7.4 PBS at the rate of 50 μL min^−1^. Then, 50 μL of different concentrations of PSA was injected at the rate of 10 μL min^−1^ with incubation for 30 min at 37 °C and washed thoroughly using 100 μL pH 7.4 PBS at the rate of 50 μL min^−1^. Furthermore, 50 μL of HRP-conjugated anti-PSA antibody (1:1000 dilution) was injected at the rate of 10 μL min^−1^, incubated for 30 min at 37 °C, and washed thoroughly using 100 μL pH 7.4 PBS at the rate of 50 μL min^−1^. Finally, we injected 50 μL of 1× pH 7.4 PBS containing 4.5 mM hydroquinone and 0.1 mM hydrogen peroxide solutions at the rate of 50 μL min^−1^. After the peak current is steady, we averaged the three measurements of current and calculated the corresponding standard deviation. At last, a chronoamperometry was implemented at the constant potential of 4 mV, in eight repeats for each group. In ensuring the CASPE-MFD to be in the best condition always during the electrochemical experiment, the electrode of CASPE-MFD was activated first by scanning within the potential range 0.5 to 1.5 V for 10 cycles in freshly prepared 0.5 M H_2_SO_4_ solutions using cyclic voltammetry. The typical voltammogram characteristic of the clean polycrystalline gold was presented. Then, the CASPE-MFD was washed with ultrapure water and PBS solutions.

## Results and Discussion

### Preparation of CASPE-MFDs

**Fig. 2:**
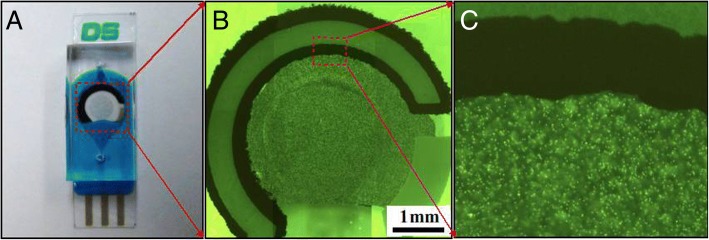
**a** Screen-printed photoelectrode used to take fluorescence images. **b** Fluorescence image of CASPE-MFD. We use a photoelectrode as a model fluorescence image to demonstrate that the working area is full with dyes and has no bubbles in the CASPE-MFD. **c** Partial enlarged drawing of the fluorescence image

Homogeneous distribution was used to investigate the utility of the CASPE-MFD. A solution of fluorescent microbeads was injected into the channels of a CASPE-MFD at a 5-μL/min flow rate, and it is obvious that every corner of the CASPE-MFD was filled with the solution of fluorescent microbeads and no bubble was formed in the device (Fig. 2). The flow rate was increased to 100 μL/min in order to prove the robustness of the CASPE-MFD, which showed that the device is suitable for analyte detection.

**Fig. 3:**
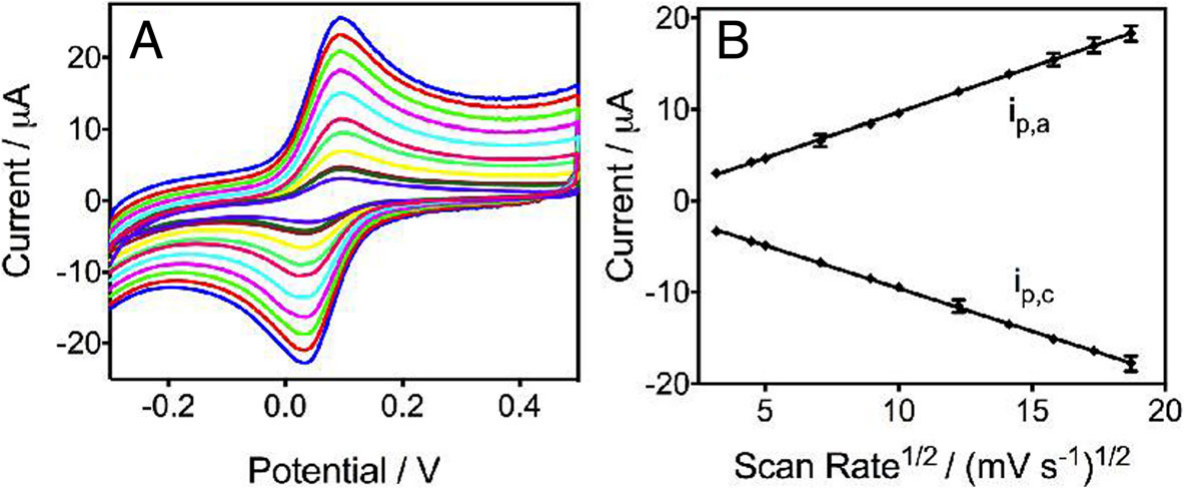
**a** Cyclic voltammograms of 0.5 mM ferrocene carboxylic acid in 0.1 M KCl aqueous solution (pH 7.0) in CASPE-MFD at different scan rates (ascending along the *y*-axis): 10, 25, 50, 80, 100, 150, 200, 250, 300, and 350 mV/s. **b** Calibration plots of the anodic (*i*_pa_) and cathodic peak current (*i*_pc_) vs the square scan rate. The two lines represent a linear curve with regression equation, respectively: *Y* (*i*_pa_) = 0.9932*X* − 0.2563 (*R*^2^ = 0.9996, *n* = 8); *Y* (*i*_pc_) = − 0.9384*X* − 0.1774 (*R*^2^ = 0.9996, *n* = 8)

The fabrication process was also investigated by cyclic voltammograms as shown in Fig. 3. Ferrocenecarboxylic acid was used as the model redox-active compound, and Fig. 3a shows the relationship of the redox peak currents with different potential scan rates. The redox peak of the CV curves exhibits a typical reversible electrochemical reaction in which the rate of reaction is governed by the diffusion of the electroactive species to the electrode surface. The potential separation between peak cathodic potential (*E*_pc_) and peak anodic potential (*E*_pa_) is 62 mV, which is close to the theoretical value of 59 mV for the ferrocene redox couple. In addition, the position of peak potentials does not alter as a function of the potential scan rates, and the anodic peak current (*i*_pa_) is approximately equal to the cathodic peak current (*i*_pc_) in the range of 10 to 350 mV/s. The reversible behavior is corresponding with the signal in bulk solution (Additional file 1: Fig. S1A), which indicates that no side reactions take place and that, as expected, the kinetics of electron transfer is sufficiently rapid to maintain the surface concentrations of redox-active species at the values required by the Nernst equation. Figure 3b shows that both anodic peak current (*i*_pa_) and cathodic peak current (*i*_pc_) were proportional to the square root of the scan rates, implying a typical diffusion-controlled process [18]. Furthermore, the current measured in CASPE-MFDs is fairly close to the value of the current in bulk solution (Additional file 1: Fig. S1B), which indicated that an analysis in the device does not sacrifice its sensitivity.

### Performance of the CASPE-MFDs on PSA Detection

**Fig. 4:**
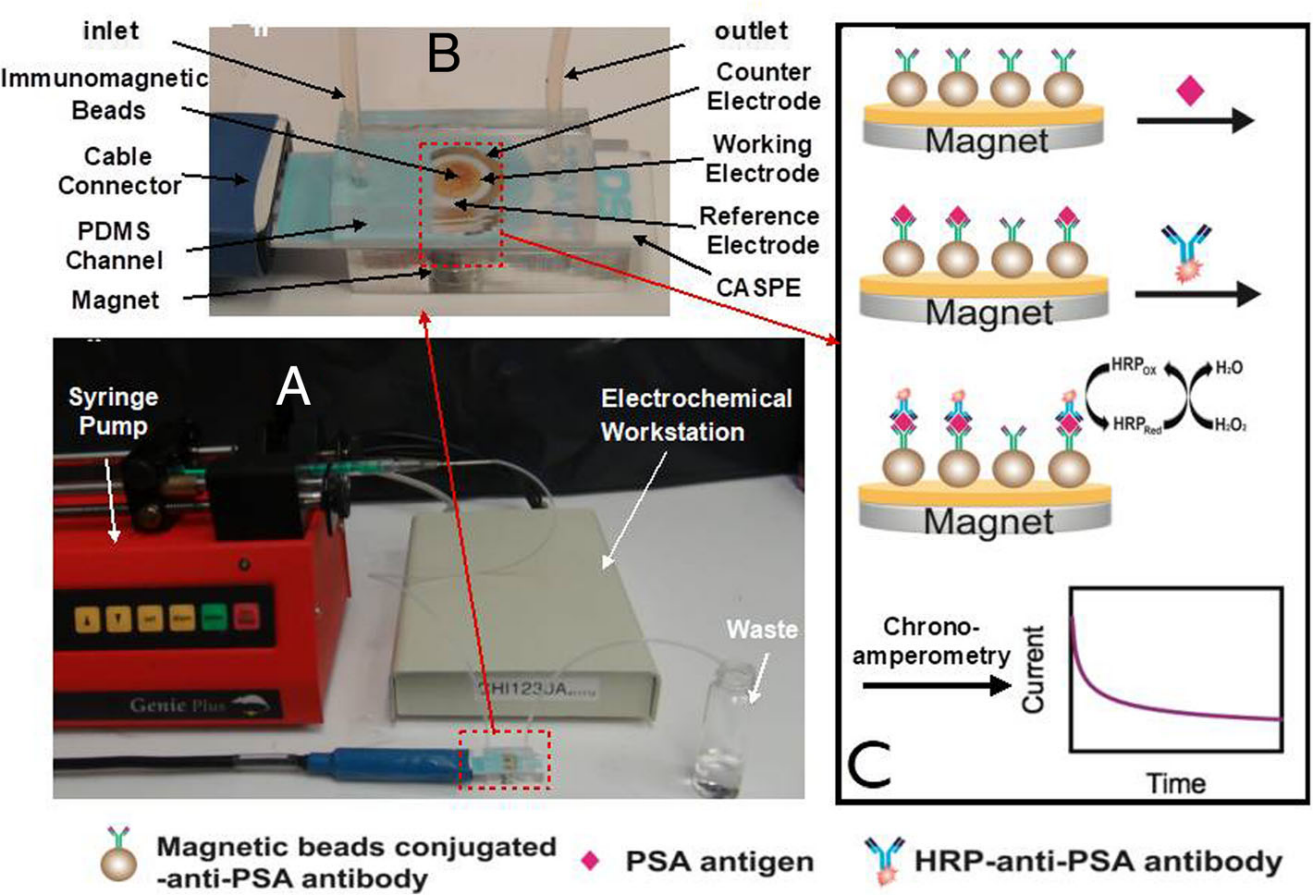
**a** The whole detection device. The syringe pump was used to inject solution into the CASPE-MFD, and the electrochemical workstation was used to detect the electrochemical signals. **b** The CASPE-MFD used to detect PSA. Immunomagnetic bead-conjugated anti-PSA antibody was injected with solutions through inlet, and a magnet was used to capture the magnetic beads. **c** Schematic of the CASPE-MFD in detection of PSA antigen. Immunomagnetic bead-conjugated anti-PSA antibody was immobilized on the working electrode using a magnet. PSA antigen was injected into the CASPE-MFD and conjugated with the anti-PSA antibody. HRP-modified anti-PSA antibody was then conjugated with PSA antigen. Chronoamperometry was used to detect the electrochemical signals that hydroquinone and hydrogen peroxide produced

Recent reports have indicated that the prostate-specific antigen (PSA) concentration in the range 4–10 ng/mL generally indicates a high probability of the presence of prostate carcinoma [19]. Therefore, PSA was chosen as a target to evaluate the performance of the prepared CASPE-MFD (Fig. 4). Figure 4a shows the prepared CASPE-MFD can be directly plug into a portable electrochemical workstation. As shown in Fig. 4c, the magnetic bead-conjugated anti-PSA antibody was immobilized on the surface of gold electrode (working electrode) using a magnet. PSA antigen was then injected into the microfluidic channels of the prepared CASPE-MFD and conjugated with the anti-PSA antibody that immobilized on the working electrode. Next, HRP-modified anti-PSA antibody was conjugated with PSA antigen. Chronoamperometry was used to detect the electrochemical signals that hydroquinone and hydrogen peroxide produced.

Chronoamperometry gives a better signal-to-noise ratio in comparison to other amperometric techniques [20–24], and the use of a thin slab of fluids mechanically clamped to the electrodes is more resistant to vibration than analysis in a larger volume of solution. For faradaic diffusion-limited currents, the current-time response is described by the Cottrell equation.

$$ i=\frac{nFA{D}^{\frac{1}{2}}C}{{\left(\pi t\right)}^{\frac{1}{2}}} $$

where *n* is the number of electrons, *F* is Faraday’s constant (96,485 C/mol), *A* is the electrode area (cm^2^), *D* is the diffusion coefficient (cm^2^/s), and *C* is the concentration (mol/cm^3^).

**Fig. 5:**
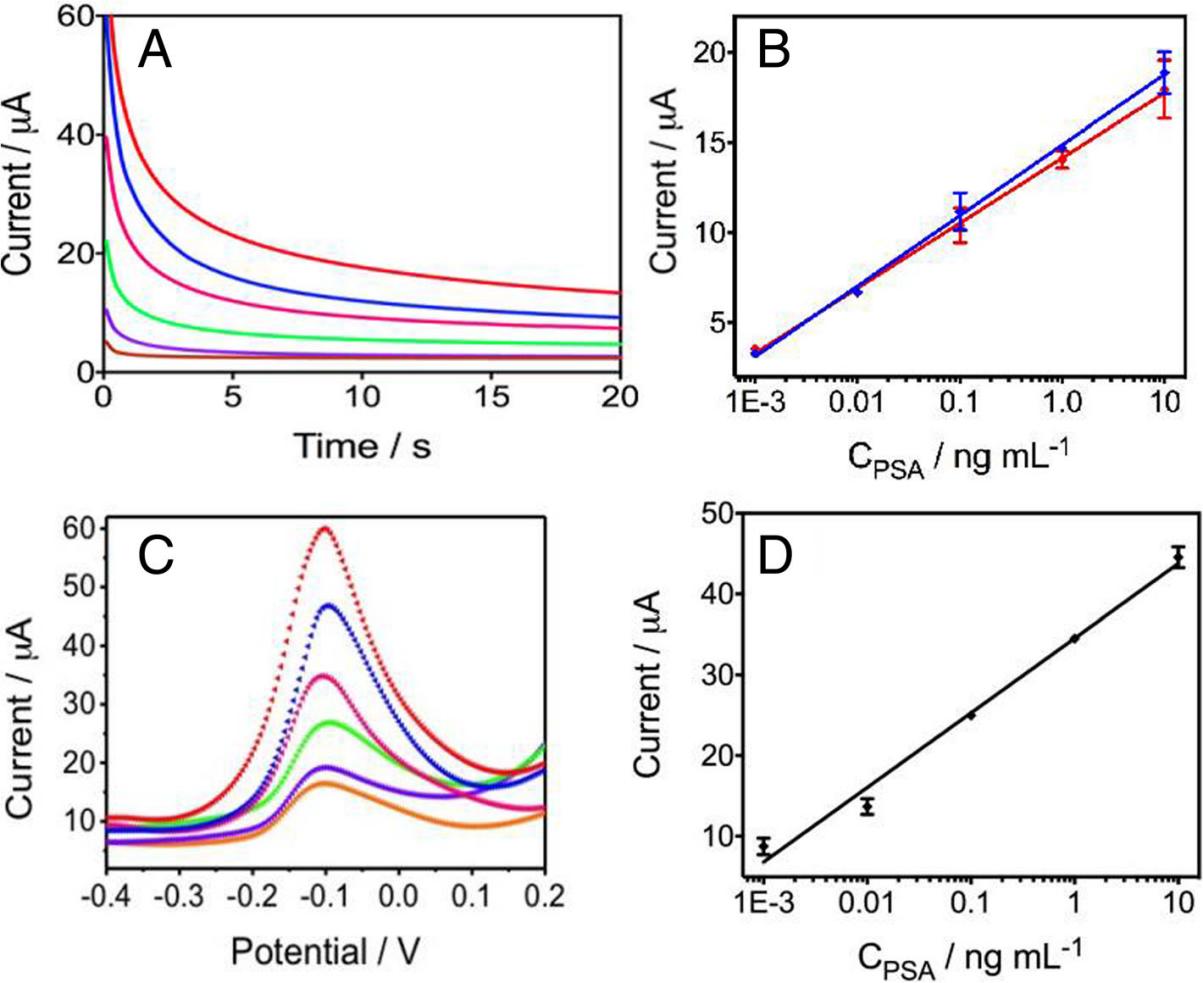
**a** Chronoamperometric curves for various concentrations of PSA antigen (ascending along the *y*-axis): 0, 0.001, 0.01, 0.1, 1, and 10 ng/mL in pH 7.4 PBS buffer containing 4.5 mM hydroquinone and 0.1 mM H_2_O_2_ solution in CASPE-MFD at − 2.0 mV vs silver pseudo-reference electrode. **b** The linear relationship between peak current and PSA antigen concentration in the CASPE-MFDs in pH 7.4 PBS buffer (blue line) and in human serum (red line). The linear regression equation of the blue line is *Y* = 14.87 + 3.927 × *X* (*R*^2^ = 0.9985, *n* = 8), and the linear regression equation of the red line is *Y* = 14.15 + 3.622 × *X* (*R*^2^ = 0.9986, *n* = 8). **c** Square wave voltammograms for various concentrations of PSA antigen in pH 7.4 PBS buffer containing 4.5 mM hydroquinone and 0.1 mM H_2_O_2_ solution in CASPE-MFD (ascending along the *y*-axis): 0, 0.001, 0.01, 0.1, 1, and 10 ng/mL, respectively. **d** The corresponding linear relationship of different concentrations of PSA antigen. The linear regression equation is *Y* = 34.53 + 9.246 × *X* (*R*^2^ = 0.9884, *n* = 8)

The prepared CASPE-MFD was used to detect PSA in a series of analyte solutions, concentration from 0 to 10 ng mL^−1^. The chronoamperometric responses of the detection for PSA in CASPE-MFDs were shown in Fig. 5a. The peak currents increased with increasing PSA concentration in pH 7.4 PBS containing 4.5 mM hydroquinone and 0.1 mM hydrogen peroxide. As shown in Fig. 5b (blue line), the peak currents were proportional to the logarithmic value of PSA concentrations over the range of 0.001 to 10 ng/mL and the linear regression equation is *I* (μA) = 14.87 + 3.927 × log *C*_PSA_ (ng/mL) (*R*^2^ = 0.9985, *n* = 8). The low limit of detection (0.84 pg/mL) and good linear relationship suggested that the prepared CASPE-MFD could be used to detect PSA in practical use. Besides, we also detected different concentrations of PSA in CASPE-MFDs using square wave voltammetry (SWV) in Fig. 5c. The SWV responses were also consistent with chronoamperometric results.

### Selective Detection of PSA with the CASPE-MFDs

To verify the possible application in our device for real samples, we analyzed various concentrations of PSA in human serum samples using chronoamperometry. The obtained results in Additional file 1: Fig. S2 demonstrated that the peak currents of the PSA also increased with the increasing PSA concentration in human serum containing 4.5 mM hydroquinone and 0.1 mM hydrogen peroxide. In addition, the corresponding calibration curve was shown in Fig. 5b (red line), and the linear regression equation is *I* (μA) = 14.15 + 3.622 × log *C*_PSA_ (ng/mL) (*R*^2^ = 0.9986, *n* = 8). It is obvious that there were almost no statistical differences between the two groups, indicating that the prepared CASPE-MFD was able to work in real samples. Furthermore, the CASPE-MFD was demonstrated that it has great selectivity to target PSA and could be used in clinical application to diagnose prostate carcinoma.

## Conclusions

We have developed a simple, low-cost, and portable commercial screen-printed electrode-based microfluidic electrochemical sensing. In addition, we have demonstrated the application of our CASPE-MFDs for the quantitative analysis of PSA in PBS buffer and in human serum samples. The measurement showed good sensitivity and reproducibility due to the device was directly fabricated on the commercial screen-printed electrodes. The CASPE-MFDs have five advantages: (i) it is lightweight, portable, multi-use; (ii) it is standardized; (iii) it has excellent reproducibility with high sensitivity and accuracy; (iv) it is easy to use and does not require professional medical personnel or complicated instruments; and (v) it allows for the integration of high-density detection systems into a small device. Besides, the use of a miniaturized potentiostat could make the CASPE-MFDs capable of field or home diagnosis. Furthermore, the commercial electrodes and easy fabrication could achieve the standardization and industrialization of the CASPE-MFDs. Therefore, we believe that this platform be widely used for point-of-care diagnosis such as small molecules (sodium, potassium, chloride, glucose), cancer markers (B-type natriuretic peptide or BNP, troponin I), cells (CD_4_), and nucleic acids (DNA, RNA).

## Additional file

Additional file 1: **Fig.S1 A**: Cyclic voltammogramms of 0.5 mM ferrocene carboxylic acid in bulk solution in CASPE-MFD at different scan rates (ascending along y-axis): 10, 25, 50, 80, 100, 150, 200, 250, 300, 350mV/s. **Fig.S1 B**: Calibration plots of the anodic (ipa) and cathodic peak current (ipc) vs the square scan rate. The two lines represent a linear curve with regression equation, respectively: Y (ipa) = 1.008X - 0.8604 (R2 = 0.9988, *n* = 8); Y (ipc) = -0.9610X - 0.0318 (R2 = 0.9998, *n* = 8). **Fig. S2**: Chronoamperometric curves for various concentrations of PSA antigen (ascending along y-axis): 0, 0.001, 0.01, 0.1, 1 and 10 ng/mL in human serum samples containing 4.5 mM hydroquinone and 0.1 mM H2O2 solution in CASPE-MFD at -2.0 mV step potential (vs. a silver pseudo-reference electrode). (PDF 208 kb)

## Abbreviations

MFDs: Microfluidic devices
CASPE-MFDs: Screen-printed electrode-based microfluidic devices
PDMS: Polydimethylsiloxane
PSA: Prostate-specific antigen
CA: Chronoamperometry
SWV: Square wave voltammetry
LOD: Limit of detection
HRP: Horseradish peroxidase
TEOS: Tetra ethoxy silane
MTES: Metastable transfer emission spectroscopy
BNP: B-type natriuretic peptide
