# Supplementary material for: Microfluidic Device Directly Fabricated on Screen-Printed Electrodes for Ultrasensitive Electrochemical Sensing of PSA
Source: Nanoscale Res Lett. 2019 Feb 28;14:71. doi: 10.1186/s11671-019-2857-6 (PMC6395463; doi:10.1186/s11671-019-2857-6)
Supplement: Supplementary file 1 — Fig.S1 A: Cyclic voltammogramms of 0.5 mM ferrocene carboxylic acid in bulk solution in CASPE-MFD at different scan rates (ascending along y-axis): 10, 25, 50, 80, 100, 150, 200, 250, 300, 350mV/s. Fig.S1 B: Calibration plots of the anodic (ipa) and cathodic peak current (ipc) vs the square scan rate. The two lines represent a linear curve with regression equation, respectively: Y (ipa) = 1.008X - 0.8604 (R2 = 0.9988, n = 8); Y (ipc) = -0.9610X - 0.0318 (R2 = 0.9998, n = 8). Fig. S2: Chronoamperometric curves for various concentrations of PSA antigen (ascending along y-axis): 0, 0.001, 0.01, 0.1, 1 and 10 ng/mL in human serum samples containing 4.5 mM hydroquinone and 0.1 mM H2O2 solution in CASPE-MFD at -2.0 mV step potential (vs. a silver pseudo-reference electrode). (PDF 208 kb) [file 11671_2019_2857_MOESM1_ESM.pdf]

Supporting Materials

Microfluidic Device Directly Fabricated on Screen-Printed Electrodes for Ultrasensitive Electrochemical Sensing of PSA

Authors: Shouhui Chen[1†], Zhihua Wang[2†], Xinyuan Cui[3], Linlei Jiang[1], Yuee Zhi[1], Xianting Ding[4], Zhihong Nie[5], Pei Zhou[1*] and Daxiang Cui[2,6*]

1, Center of Food Safety Engineering and Technology Research, Shanghai, Key Laboratory of Urban Agriculture, Ministry of Agriculture, School of Agriculture and Biology, Shanghai Jiao Tong University, 800 Dongchuan Road, Shanghai 200240, China.

2, Institute of Nano Biomedicine and Engineering, Shanghai Engineering Research Center for Intelligent Instrument for Diagnosis and Therapy, Key Lab. for Thin Film and Microfabrication Technology of Ministry of Education, Department of Instrument Science and Engineering, School of Electronic Information and Electrical Engineering, Shanghai Jiao Tong University, 800 Dongchuan Road, Shanghai 200240, China.

3, Department of Medical Imaging, Second Clinical College of Chongqing Medical University, No.74 Linjiang Road, Yuzhong District, Chongqing 400016, China.

4, Institute for Personalized Medicine, School of Biomedical Engineering, Shanghai Jiao Tong University, Shanghai 200030, China.

5, Department of Chemistry and Biochemistry, University of Maryland, College Park, MD 20742, USA.

6, National Center for Translational Medicine, Collaborative Innovational Center for System Biology, Shanghai Jiao Tong University, 800 Dongchuan Road, Shanghai 200240, China.

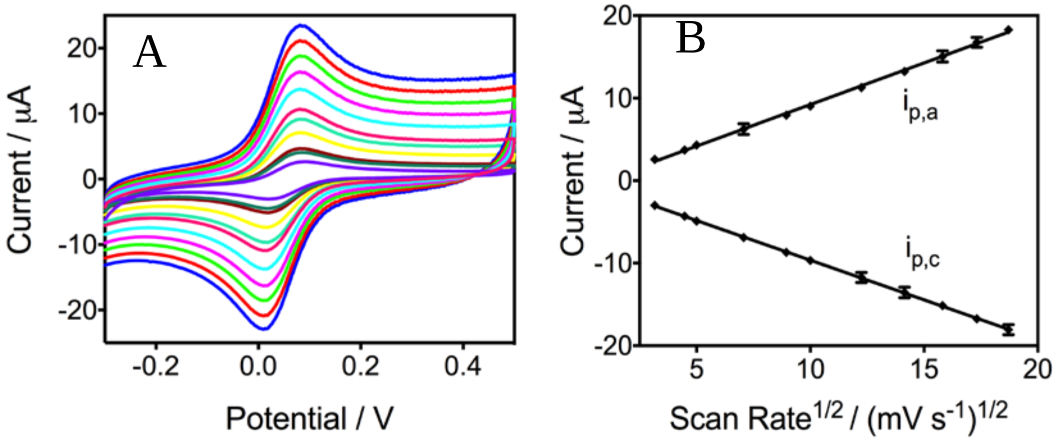

Fig. S1. (A) Cyclic voltammogramms of 0.5 mM ferrocene carboxylic acid in bulk solution in CASPE-MFD at different scan rates (ascending along y-axis): 10, 25, 50, 80, 100, 150, 200, 250, 300, 350mV/s. (b) Calibration plots of the anodic (i_{pa}) and cathodic peak current (i_{pc}) vs the square scan rate. The two lines represent a linear curve with regression equation, respectively: Y (i_{pa}) = 1.008X - 0.8604 (R^2 = 0.9988, n = 8); Y (i_{pc}) = -0.9610X - 0.0318 (R^2 = 0.9998, n = 8).

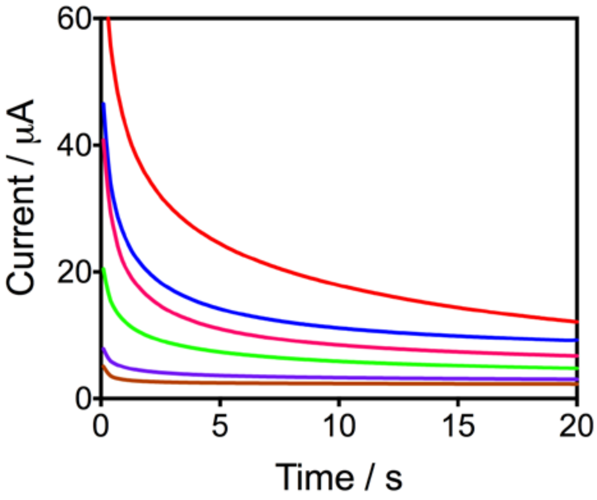

Fig. S2 Chronoamperometric curves for various concentrations of PSA antigen (ascending along y-axis): 0, 0.001, 0.01, 0.1, 1 and 10 ng/mL in human serum samples containing 4.5 mM hydroquinone and 0.1 mM H_2O_2 solution in CASPE-MFD at -2.0 mV step potential (vs. a silver pseudo-reference electrode).
